# Initial FDG-PET/CT predicts survival in adults Ewing sarcoma family of tumors

**DOI:** 10.18632/oncotarget.20335

**Published:** 2017-08-18

**Authors:** Bastien Jamet, Thomas Carlier, Loic Campion, Emmanuelle Bompas, Sylvie Girault, Fanny Borrely, Ludovic Ferrer, Maxime Rousseau, Yann Venel, Françoise Kraeber-Bodéré, Caroline Rousseau

**Affiliations:** ^1^ Nuclear Medicine Unit, ICO Cancer Center Gauducheau, Saint Herblain, France; ^2^ Nantes-Angers Cancer Research Center, INSERM U892, CNRS UMR 6299, University of Nantes, Nantes, France; ^3^ Nuclear Medicine Unit, University Hospital, Nantes, France; ^4^ Oncology Unit, ICO Cancer Center Gauducheau, Saint Herblain, France; ^5^ Nuclear Medicine Unit, ICO Cancer Center Papin, Angers, France; ^6^ Nuclear Medicine Unit, University Hospital Bretonneau, Tours, France; ^7^ Physics Unit, ICO Cancer Center Gauducheau, Saint Herblain, France

**Keywords:** FDG-PET/CT, oncology, Ewing sarcoma family of tumors, survival analysis, prognosis

## Abstract

**Purpose:**

The aim of this retrospective study was to determine, at baseline, the prognostic value of different FDG-PET/CT quantitative parameters in a homogenous Ewing Sarcoma Family of Tumors (ESFT) adult population, compared with clinically relevant prognostic factors.

**Methods:**

Adult patients from 3 oncological centers, all with proved ESFT, were retrospectively included. Quantitative FDG-PET/CT parameters (SUV (maximum, peak and mean), metabolic tumor volume (MTV) and total lesion glycolysis (TLG) of the primary lesion of each patient were recorded before treatment, as well as usual clinical prognostic factors (stage of disease, location, tumor size, gender and age). Then, their relation with progression free survival (PFS) and overall survival (OS) was evaluated.

**Results:**

32 patients were included. Median age was 21 years (range, 15 to 61). Nineteen patients (59%) were initially metastatic. On multivariate analysis, high SUV_max_ remained independent predictor of worst OS (p=0.02) and PFS (p=0.019), metastatic disease of worst PFS (p=0.01) and high SUVpeak of worst OS (p=0.01). Optimal prognostic cut-off of SUV_peak_ was found at 12.5 in multivariate analyses for PFS and OS (*p*=0.0001).

**Conclusions:**

FDG-PET/CT, recommended at ESFT diagnosis for initial staging, can be a useful tool for predicting long-term adult patients outcome through semi-quantitative parameters.

## INTRODUCTION

The term Ewing sarcoma family of tumours (ESFT) indicates a family of morphologically similar malignancies that includes classic Ewing sarcoma of bone, extraskeletal Ewing sarcoma, small cell tumour of the thoraco-pulmonary region (Askin tumour), and soft tissue-based primitive neuroectodermal tumours (PNET) [1]. It is an aggressive sarcoma of bone and/or soft tissue with a peak incidence during adolescence and young adulthood [2] and the second most common primary bone tumour [3].

Clinically relevant prognostic factors (age, tumor size, location, male gender and metastatic disease) were identified by international studies conducted over the last three decades and confirmed in a large and recent epidemiologic study [4]. With advances of multimodal therapy (chemotherapy and surgery with or without radiotherapy), survival has improved for patients with localized disease [4, 5] but those with a metastatic disease still have a poor prognosis with a five-year overall survival (OS) of 30% [6, 7]. However, patients with isolated pulmonary metastasis have a better clinical outcome than those with metastases at other sites [6, 8]. For localized tumors resected after induction chemotherapy, histologic response is the strongest independent prognostic factor, regardless of the grading system used [9, 10].

Before using ^18^F-fluorodeoxyglucose positron emission tomography with computed tomography (FDG-PET/CT) for ESFT staging, imaging for this pathology management included only MRI (local extent tumor evaluation) and CT associated with bone scan for metastatic status. As with other malignancies, then the role of FDG-PET/CT is now well established for the staging of primary and recurrent ESFT [11–14]. In a meta-analysis, Treglia *et al*. [11] showed at staging a pooled sensitivity of 96% and a pooled specificity of 92%. FDG-PET/CT can detect practically 100% of the primary tumors (as other conventional imaging methods) but is superior in the detection of bone metastases (sensitivity of 88% versus 37% for bone scan) [14]. Conversely, FDG-PET/CT seems to be less sensitive than CT for the depiction of small lesions, mainly represented by pulmonary metastases due to lowest spatial resolution and spontaneous breathing during the exam [15, 16]. Therefore, the combination of FDG-PET/CT with morphological imaging is a valuable tool for the staging and restaging of ESFT and has a relevant impact on the treatment strategy plan [17]. Moreover, recent studies (with mixed paediatric and adult populations) showed that FDG-PET/CT seemed to have a prognostic value for PFS after neoadjuvant chemotherapy [18–20] and FDG-PET/CT changes in metabolic activity of the primary tumor seemed to be correlated with histopathological response [21]. In addition, at diagnosis, Raciborska *et al*. [19] also showed in a series of 50 paediatric patients that FDG-PET/CT SUV_max_ was significantly lower for patients with good histological response than for patients with poor histological response.

Nevertheless, few data on the prognostic value of FDG-PET/CT at baseline has been released. Only a recent retrospective study with a mixed children and adults cohort [22], showed that SUV_max_>5.8 was an independent factor associated with worse overall survival.

The aim of this retrospective study was to determine, at baseline, the prognostic value of different FDG-PET/CT quantitative parameters in a homogenous ESFT adult population compared with clinically relevant prognostic factors.

## RESULTS

### Patient characteristics

Clinical characteristics of the 32 patients are listed in Table 1. All patients had histological confirmed ESFT at the time of diagnosis. Most of patients (19/32: 59%) had metastatic disease at diagnosis: 8/32 (25%) in lungs, 8/32 (25%) in bone and 3/32 (9%) in both locations. During follow-up, eighteen of the 32 patients (56%) have experienced metastatic disease recurrence; eight of them (25%) died specifically from their disease.

**Table 1:** Patient characteristics

Variable	N	%
**Patients**	32	
Median age (range)	21 (15 to 61)	
**Type of primary lesion**		
Bone	21	66
Extra-skeletal	11	34
**Gender**		
Male	18	56
Female	14	44
**Primary tumor location**		
Axial	18	56
Peripheral	14	44
**Size**		
>10 cm	16	50
<10 cm	16	50
**Stage of disease**		
Metastatic	19	59
Localized	13	41
**Metastatic sites**		
Lungs	8	25
Bones	8	25
Lungs and bones	3	9

ES: Ewing Sarcoma.

Response to the neoadjuvant chemotherapy, assessed by pathological analysis, was performed for 18/32 (56%) patients. Median follow-up was 32 months (range, 2 to 64) and median time to relapse (or progression) was 18 months (range, 9 to 56).

### Univariate analyses

The 3-years PFS and OS survival of the population were estimated to 35% and 63%, respectively. Results of univariate survival analyses are presented in Table 2. Male gender and metastatic disease were associated with a significant worse PFS but neither of them adversely affected OS. In particular, metastatic disease had a trend toward reduced OS but no statistical significance (metastatic patients had 3-years OS of 43% vs. 84% for non-metastatic patients, p = 0.149). There was not significant association between outcome and other clinical parameters as age, location, tumor size.

Table 2A

**Table:** 

Variables	*N*	3-yr PFS(%) [95%CI]	p	3-yr OS(%) [95%CI]	p
**Gender**					
Female	14	62 [26–84]		85 [52–96]	
Male	18	14 [2–35]	0.01	47 [20–71]	0.19
**Stage of disease**					
Localized	13	73 [37–90]		84 [49–96]	
Metastatic	19	7 [1–28]	0.0004	43 [15–69]	0.15
**SUV_max_**					
<17	26	41 [20–60]		72 [44–87]	
≥17	6	0 [0–0]	0.001	21 [1–60]	0.004
**SUV_peak_**					
<12.5	26	36 [17–56]		72 [44–87]	
≥12.5	6	0 [0–0]	0.002	0 [0–0]	<0.001
**SUV_mean40%_**					
<8.5	25	44 [23–63]		71 [43–87]	
≥8.5	7	0 [0–0]	0.017	34 [5–69]	0.018

SUV: Standardized Uptake Value; PFS: Progression Free Survival; OS: Overall Survival; HR: Hazard Ratio; CI: Confidence Interval.

**Table:** B

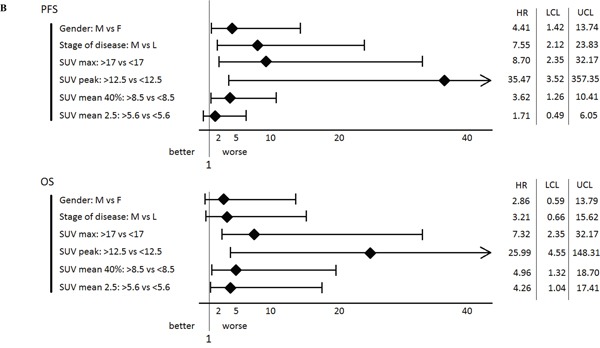

For FDG-PET/CT-derived quantitative parameters, the degree of FDG uptake at baseline was an unfavorable prognostic factor. Indeed, high SUV_max_, SUV_peak_, and SUV_mean40%_ significantly adversely affected PFS and OS. An additional parameter influenced only OS: high SUV_mean2.5_ value which shortened survival. Discretization of SUV_max_ showed that 3-years PFS and OS survival of patients with baseline SUV_max_≥17 were decreased compared to patient with lower SUV_max_ (OS: 21% vs 72%, respectively; p=0.004; PFS: 0% vs 41%, respectively; p=0.001). Similarly, discretization of SUV_peak_ showed that 3-years PFS and OS survival of patients with baseline SUV_peak_≥12.5 were significantly decreased compared to patients with lower SUV_peak_ (OS: 0% vs 72%, respectively; p<10^-3^; PFS: 0% vs 36%, respectively; p=0.002).

There was not statistically significant association between outcome (PFS or OS) and primary lesion functional volumes (MTV or TLG) even if various thresholds were tested.

### Multivariate analyses

**Figure 1:**
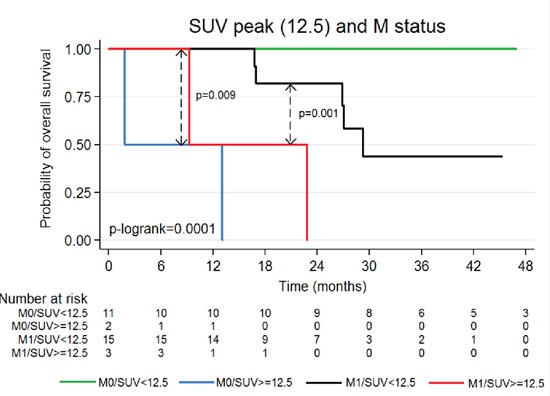
OS according to SUV_peak_ and metastatic status (N=31) (Too small tumor for one patient to quantify with SUV_peak_)

**Figure 2:**
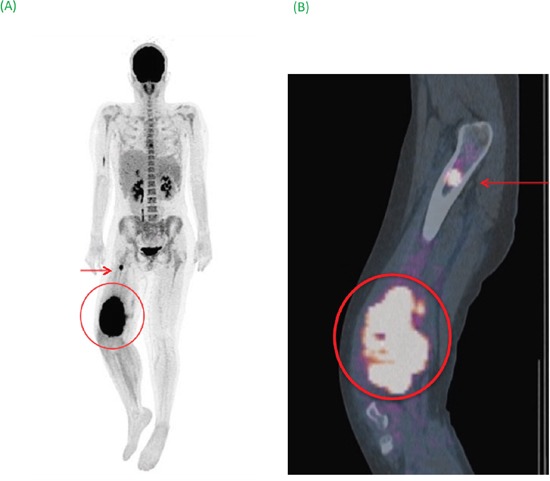
**(A)** Maximum image projection (MIP) showed the primary tumor surrounded associated with bone metastatic site (arrow). **(B)** Sagittal fusion FDG-PET/CT image specified lesions’ anatomical locations. Semi-quantitative PET parameters of the primary tumor (SUV_max_ of 29 and SUV_peak_ of 20.57) were associated with a poor prognosis (OS: 9 months).

Results of multivariate survival analyses are presented in Table 3 for PFS and Table 4 for OS. In summary, high SUV_max_value was an independent and unfavorable factor for PFS and OS (p=0.019) and (p=0.02) respectively. Moreover, high SUV_peak_ value was an independent and unfavorable factor for OS (p=0.01) but only had a limited prognostic value for PFS (p=0.057). PFS was also negatively and independently influenced by metastatic status at baseline, which is not observed at the analysis for OS. Optimal prognostic cut-off (CO) for SUV_peak_ was determined as 12.5 for PFS and OS (p=0.0001). Kaplan–Meier curves of OS according to FDG-PET/CT-SUV_peak12.5_ measurement at baseline and disease stage are depicted in Figure 1. Metastatic patients with SUV_peak_≥12.5 had at 18-months an estimate of OS of 50% vs. 82% if SUV_peak_<12.5 (p=0.001), as illustrated with Figure 2. In the non-metastatic sub-group of patients, all patients with SUV_peak_≥12.5 died during the first fifteen months of follow-up, while none of those with SUV_peak_<12.5 (p =0.009) died.

**Table 3:** Multivariate Cox regression models for the progression free survival analysis

Models	Multivariate analysis	
	Hazard Ratio (95% CI)	*p*
**Model 1**		
SUV_max_	1.12 (1.02-1.24)	0.019
Metastatic disease	5.83 (1.52-22.46)	0.01
Male gender	2.8 (0.82-9.59)	0.1
**Model 2**		
SUV_peak_	1.17 (0.99-1.38)	0.057
Metastatic disease	6.08 (1.56-23.67)	0.009
Male gender	3.19 (0.91-10.98)	0.07

CI: Confidence Interval; SUV: Standardized Uptake Value.

**Table 4:** Multivariate Cox regression models for the overall survival analysis

Models	Multivariate analysis	
	Hazard Ratio (95% CI)	*p*
**Model 1**		
SUV_max_	1.17 (1.02-1.34)	0.02
Metastatic disease	1.62 (0.29-9.01)	0.58
Male gender	2.23 (0.39-12.59)	0.36
**Model 2**		
SUV_peak_	1.31 (1.05-1.65)	0.01
Metastatic disease	1.75 (0.32-9.47)	0.51
Male gender	2.18 (0.38-12.29)	0.37

CI: Confidence Interval; SUV: Standardized Uptake Value.

**Figure 3:**
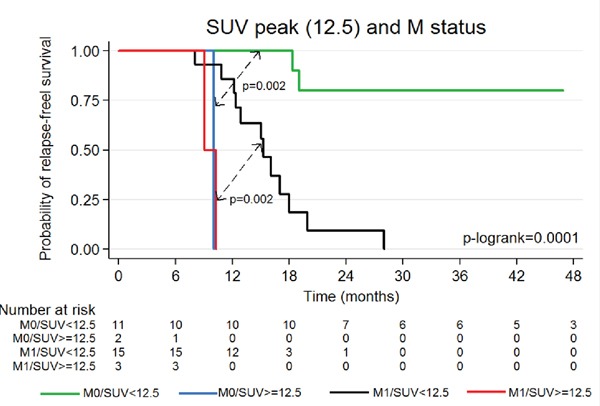
PFS according to SUV_peak_ and metastatic status (N=31) (Too small tumor for one patient to quantify with SUV_peak_)

Kaplan–Meier curves of PFS according to FDG-PET/CT-SUV_peak_ measurement at baseline and disease stage are depicted in Figure 3. All metastatic patients with SUV_peak_≥12.5 relapsed or progressed during the first 12 months of follow-up. Conversely, patients with SUV_peak_<12.5 had a 1-year PFS survival of 86% (p=0.002). In the non-metastatic sub-group of patients, all patients with SUV_peak_≥12.5 relapsed or progressed during the first 12 months of follow-up, while those with SUV_peak_<12.5 had 2-years PFS survival of 80% (p=0.002).

None optimal prognostic cut off of SUV_max_was found in multivariate analysis for the PFS and OS in this cohort.

## DISCUSSION

Recently, Hwang *et al*. [22] has showed, for the first time, the prognostic value of FDG-PET/CT at diagnosis in ESFT patients, but in a mixed population (children and adults), as for all studies about this disease [11, 18, 19, 22].

To our knowledge, our study is the first to explore prognostic value of FDG-PET/CT at diagnosis for exclusively ESFT adult patients.

One major result can be highlighted: in a homogeneous adult cohort, treated according to the same treatment protocol, FDG-PET/CT's semi-quantitative parameters as SUV values were strong predictors of clinical outcomes as early as at diagnosis. This is the key point of our study and highlights a new role of the FDG –PET/CT in the management of adult ESFT patients.

A meta-analysis about 2 studies (patients with soft tissue sarcomas) suggested that FDG-PET/CT at diagnosis provides a very useful prognostic OS tool for patients through the SUV [27]. But these studies assessed mixed cohorts (age and histological results differed), allowing only a careful interpretation of the FDG-PET/CT semi-quantification. Hwang study demonstrated that patient age and metastatic status were found to be independent predictors of overall survival, [4, 10, 22, 28, 29]. Whether the age of the patient is an independent prognostic factor should focus our attention on the question of the relevance of mixing adult and pediatric patients in cohorts for this type of pathology. The disease appeared less unfavorable if patient age was <20 years, raising the possibility of a different disease according to the patients age. In our study, with adults exclusively, patient age was not found as a prognostic factor (certainly due to the population homogeneity). In contrast, metastatic status remained as an independent prognostic factor for the PFS (p=0.01). This is probably due to the high prevalence of metastatic patients (nearly 60%), to the low incidence of death during follow-up (9/32) and the limited number of patients. Nevertheless, this clinical parameter is well known as the strongest adverse prognostic factor [6, 8].

The second interesting point in Hwang study was the SUVmax cut off of 5.8 as an independent prognostic factor for OS [22], contrary to Hawkins et al. [18] study findings. However, populations for these two studies concerned a mixed (adult and pediatric) recruitment. For our part, in exclusively adult population, in multivariate analysis, high SUVmax value was an independent and unfavorable factor for OS (p=0.02) but no discriminating cut off was found.

Our multi-parameters study (SUV_max,_ SUV_peak_, SUV_mean2.5_, SUV_mean40%,_ MTV_2.5_, MTV_40%_, TLG_2.5_, TLG_40%_), found two unfavorable independent prognostic parameters for the OS and PFS: the SUV_max_ without individualized cut-off and the SUV_peak_ with an optimal cut-off determined at 12.5. The SUV_max_, mainly used in clinical practice, has the disadvantage of being affected by the image noise [30]. In our study, primary ESFT are usually large and very avid to FDG giving a high tumor/background noise ratio allowing clinicians to overcome this limitation. The use of SUV_peak_ was recommended as a more robust alternative due to its fixed volume of 12 mm diameter, less subject to noise than SUV_max_ [31]. The reproducibility of SUV_peak_ is less affected by acquisition equipment or pixel size changes as could be occurred in multicentric studies. This property could have advantages for multicenter studies, in which images from different sites are likely to have pixels of different sizes. This parameter is the most significant of our study with the ability to determine an optimal cut-off for both the OS and PFS. The SUV_mean_ parameter, whatever the segmentation approach used, was not an independent prognostic factor in our study and we can assume that the method of calculating the SUV_mean_, by averaging data from several pixels, is responsible of a loss of information, especially regarding the most intense pixels which most likely correspond to the most aggressive cell clusters.

The potential clinical impact of using parameters as MTV or TLG, reflecting overall tumor metabolic information rather than a single or few voxel measures based on SUV_max_ or SUV_peak_ has been recently demonstrated [32]. MTV and TLG seem to be useful for the FDG-PET/CT's therapeutic response evaluation of various tumors [33, 34] and high MTV or TLG has an unfavorable prognostic value at baseline in many solid tumors like breast [35], lung [36] or esophageal cancers [37]. In ESFT patients, one study concerning neoadjuvant chemotherapy assessment showed that a significant decrease of MTV during treatment was associated with favorable histologic response [21]. We were interested on relation with survival of MTV's and TLG's primary tumor at baseline for the first time at our knowledge, but these parameters were not prognostic factors in our study. The TLG is unlikely because of its definition corresponding to the combination of two parameters (SUV_mean_ and MTV) not prognostic in our study. For MTV, one can hypothesize that the prognosis is possibly less impacted by MTV than the detection of the most intense pixels (SUV_max_, SUV_peak_), that correspond to the most aggressive cell clusters with prognostic consequence.

The limitation of our study is its retrospective design associated with the small number of patients. However, the fact the strongest prognostic factor was SUV_peak_ instead of SUV_max_ makes the general applicability of our study more transferable to other centers. The multi-centric design of semi-quantitative data analysis could be a problem for the robustness of the results of this study. But the most important semi-quantitative parameters that are significant in this study are the SUVpeak and SUV max slightly impacted by the partial volume effect. As suggested by Visvikis et al. SUV_max_ is in principle less dependent to partial volume effects (PVE), resulting from the limited spatial resolution of PET imaging. SUV_peak_ can reduce the SUV_max_ sensitivity to noise and this decreases the variance of the PET/CT results [32, 38]. Kelly et al. showed that despite the use of different reconstruction algorithms, the SUV_peak_ remains within the limits of the max and min recovery coefficients of European best practice guides [38].

## MATERIALS AND METHODS

### Patients

This retrospective study assessed exclusively adult patients with ESFT who received treatment according to the Euro-Ewing 99 protocol [6, 23] during the period 2010-2015, in 3 oncological centers. All patients had histological confirmation of Ewing sarcoma at diagnosis with surgical biopsy and (at the same time) underwent local extent primary tumor evaluation with MRI complemented with thoraco-abdomino-pelvian CT and FDG-PET/CT for metastatic assessment before treatment. Metastatic status was confirmed by biopsies only in equivocal cases.

For each patient, at baseline, recognised clinical prognostic factors for survival [4] as: age, gender, primary tumor location (axial or peripheral), tumor size (≥10 or <10cm), stage of disease (metastatic or localized), metastatic sites (lungs only, bone only or both sites), were collected.

All patients received before primary tumor surgery a neoadjuvant phase with Vincristine, Ifosfamide, Doxorubicin, and Etoposide (VIDE, 6 courses). Treatment response was assessed by pathological analysis with the percentage of viable tumor cells remaining after neoadjuvant chemotherapy. Good response was defined as ≥90% necrosis, and poor response was defined as<90% necrosis, according to the Huvos-derived Salzer-Kuntschik grading system [9, 24]. Patients with a good histological response received adjuvant chemotherapy with Vincristine, Dactinomycin, and Cyclophosphamide/Ifosfamide (VAI/VAC). Patients with a poor primary tumor histological response received a consolidation phase with high-dose therapy (Busulfan and Melphalan regimen) followed by hematopoietic stem cell rescue. Patients with persistent metastasis before surgery underwent radiotherapy associated to adjuvant chemotherapy (VAI/VAC). Clinical follow-up was identical in the 3 oncological centers. Relapse was clinically suspected and documented by MRI and/or CT +/- FDG-PET/CT.

All patients received written information and we obtained informed consent from all of them allowing the use of their clinical data for research purposes under a protocol approved by the ethics committee.

### FDG-PET/CT acquisition

After 6 h of fasting, 3MBq/kg of 18F-FDG was injected intravenously (after recording blood glucose level). After an average of 62+/-5min of resting, whole body FDG-PET/CT imaging was recorded in supine position from the skull to the toes with arms at sides. Images were acquired on a Siemens Biograph mCT (n = 27), Philips Gemini (TF16, n = 3) and GEMS Discovery ST (n = 2). First, CT acquisition was performed with a multislice spiral CT scanner (dose modulation with a quality reference of 120kV, 80mAs, 3 mm slice thickness and a pitch of 0.75). Then, a whole body PET acquisition of the same axial range was done with the patient in the same position.

### FDG-PET/CT visual analysis

Besides the primary tumor, any FDG foci that could not be explained by physiological uptake, benign disease or traumas, was considered as metastasis.

Pulmonary lesions showing the characteristic appearance of metastasis on CT scan (well-circumscribed rounded lesions with soft tissue attenuation, location in the periphery of the lung, absence of calcification, multiple nodules of variable size, ‘canon ball’ opacities, presence of ‘feeding vessel sign’) [13] were taken as positive even if no FDG uptake was observed.

### FDG-PET/CT quantitative analysis

The maximum, peak, and mean SUV values (SUV_max,_ SUV_peak_, SUV_mean_), as well as MTV (metabolic tumor volume) and TLG (total lesion glycolysis), were calculated on primary tumor. SUV_peak_ was the maximum value inside the boundary of the tumor of the mean SUV calculated within a 1 cm^3^ sphere. The SUV_mean_, MTV and TLG of primary tumor were measured using PlanetOnco (Dosisoft, France). MTV was measured with different segmentation techniques: fixed at 2.5 (MTV_2.5_) and 40%SUVmax (MTV_40%)_ thresholds (25, 26). SUVmean measured in the derived MTVs are designated as SUV_mean2.5_ and SUV_mean40%_. TLG is the product of MTV and SUVmean.

### Statistical analyses

Overall survival (OS) was measured from date of FDG-PET/CT to specific death and progression free survival (PFS) was the time interval from date of FDG-PET/CT to relapse or progression disease; survivors were censored at the time of last contact.

Correlation between primary maximal tumor diameter and MTV (2.5 and 40%) was calculated by Spearman rho. We tested the possible correlation between PFS, OS and FDG-PET/CT-derived quantitative parameters (SUV_max,_ SUV_peak_, SUV_mean2.5_, SUV_mean40%_, MTV_2.5_, MTV_40%_, TLG_2.5_, TLG_40%_), as well as international clinical prognostic parameters registered at baseline (age, tumor size, location, gender and stage of disease).

The Kaplan-Meier method was used to estimate OS and PFS with group comparisons made using the log-rank test. Univariate and multivariate analyses of imaging and clinical parameters were carried out using Cox regression model.

As SUV_max_ and SUV_peak_ are highly correlated, a multivariate analysis was performed using SUV_max_ and SUV_peak_ separately with significant clinical parameters in univariate analysis (gender and metastatic disease status only) (model 1 and 2). Optimal cut offs were assessed using the « findcut » SAS macro made from method of Contal and O'Quigley (1999) and FDR q-values were calculated.

All tests were two-sided and P-value < 0.05 was considered to indicate statistical significance.

Statistical analyses were performed using Stata 13.1 SE (StataCorp, College Station, Texas, USA). Quantitative values were expressed as mean ± standard deviation or median and range as appropriate.

## CONCLUSION

FDG-PET/CT, already recommended at ESFT diagnosis for initial staging, can also be an useful tool for predicting long-term outcome in adult patients through semi-quantitative parameters as SUV_max_ and SUV_peak_. Further research is warranted to confirm these results.
